# Algebraic invariants of the edge ideals of whisker graphs of cubic circulant graphs

**DOI:** 10.1016/j.heliyon.2025.e41783

**Published:** 2025-01-10

**Authors:** Mujahid Ullah Khan Afridi, Ibad Ur Rehman, Muhammad Ishaq

**Affiliations:** School of Natural Sciences, National University of Sciences and Technology Islamabad, Sector H-12, Islamabad, Pakistan

**Keywords:** primary, 13C15, secondary, 13F20, 05C38, 05E99, Monomial ideal, Regularity, Depth, Stanley depth, Projective dimension, Whisker graph, Cubic circulant graph

## Abstract

Let *Q* be a polynomial ring over a field F and I be an edge ideal associated with the whisker graph of a cubic circulant graph. We discuss the regularity, depth, Stanley depth, and projective dimension of Q/I.

## 1 Introduction

Let $Q:=F[u_{1},\ldots ,u_{m}]$ be a polynomial ring over a field F equipped with the standard grading and **N** be a finitely generated graded *Q*-module. Suppose **N** admits the following minimal free resolution:

$$0⟶\;\underset{j\in Z}{⨁}Q{\left(-j\right)}^{\beta_{r,j}(N)}⟶\underset{j\in Z}{⨁}Q{\left(-j\right)}^{\beta_{r-1,j}(N)}⟶\ldots ⟶\underset{j\in Z}{⨁}Q{\left(-j\right)}^{\beta_{0,j}(N)}⟶N⟶\;0.$$

The *Castelnuovo-Mumford regularity* (or *regularity*) of **N**, denoted reg(N), is defined as $\mathrm{reg}(N)=\max\{j-i:\beta_{i,j}(N)\neq 0\}$. The *projective dimension* of **N**, denoted pdim(N), is defined as $\mathrm{pdim}(N)=\max\{i:\beta_{i,j}(N)\neq 0\}$. Let G:=(V(G),E(G)) be a graph with vertex set $V(G)=\{u_{1},\ldots ,u_{m}\}$ and edge set E(G). All graphs considered in this paper are finite and simple. The *edge ideal* I(G), associated with a graph *G* is a squarefree monomial ideal, that is, $I(G)=(u_{i}u_{j}:\{u_{i},u_{j}\}\in E(G))$. For n≥2, the graph $P_{n}$ is called a *path* on *n* vertices if $E(P_{n})=\{\{u_{i},u_{i+1}\};1\leq i\leq n-1\}$. For n≥3, a *cycle* $C_{n}$ is a graph on *n* vertices with $E(C_{n})=\{\{u_{i},u_{i+1}\};\;1\leq i\leq n-1\}⋃\{u_{1},u_{n}\}$. A graph *T* is a *tree* if any two vertices of *T* are connected by a unique path. The *degree* of a vertex in a graph *G* is the number of edges incident to that vertex. A vertex of degree one in a graph is referred to as a *pendant vertex* or *leaf*, and an *internal vertex* is a vertex that is not a leaf. Let $\text{b}=(b_{1},b_{2},\ldots ,b_{m})$, where $b_{i}$ is a positive integer for each *i*, the graph W(G,b) is called the *whisker graph of G* if $b_{i}$ new pendants (whiskers) are added to the vertex $u_{i}$ for all *i*. If $b_{1}=b_{2}=\ldots =b_{m}=q$, then we represent W(G,b) by $W_{q}(G)$. A graph *G* is called *p-regular* if the degree of every vertex in *G* is *p*. For x∈R, ⌊x⌋=max⁡{a∈Z:a≤x} and ⌈x⌉=min⁡{a∈Z:a≥x}. Let I be a monomial ideal, if G(I) represents the minimal set of monomial generators of I, then $\mathrm{supp}(I):=\{\;u_{i}:u_{i}|u\text{ for some }u\in G(I)\}$. Let n≥2 and $S\subset \{1,\ldots ,\lfloor \frac{n}{2}\rfloor \}$. A *circulant graph* denoted by $C_{n}(S)$ is a graph such that $\left\{u_{i},u_{j}\}\in E(C_{n}(S)\right)$ if and only if |i−j| or n−|i−j| is an element of *S*. We denote the graph $C_{n}(\{a_{1},\ldots ,a_{r}\})$ by $C_{n}(a_{1},\ldots ,a_{r})$. A circulant graph $C_{n}(a_{1},\ldots ,a_{r})$ is 2*r*-regular, except in the case if $2a_{r}=n$, in which case it is (2r−1)-regular. Consequently, a 3-regular circulant graph has the form $C_{2n}(a,n)$, where 1≤a<n. A 3-regular circulant graph is also called a cubic circulant graph. Circulant graphs find applications in network theory [3], [20], group theory [2], and in the realm of designs and error-correcting codes [33].

Various algebraic invariants and properties of the edge ideals of circulant graphs have been studied, as evident from [4], [26], [27], [32], [34], [35], [38], [39]. The regularity of the edge ideals of cubic circulant graphs is studied by Uribe-Paczka et al. [38], and Shaukat et al. study the algebraic invariants namely depth, Stanley depth, and projective dimension of the edge ideals associated with cubic circulant graphs [34]. Wang et al. [41] compute the values of some algebraic invariants of the whisker graphs of some circulant graphs. The aim of this paper is to discuss the algebraic invariants namely depth, Stanley depth, regularity, and projective dimension associated with the edge ideals of $W(C_{2n}(a,n),\text{b})$. The following theorem plays a vital role in our main findings.

Theorem 1.1 **[10]**

*Let*1≤a<n*and*t=gcd⁡(2n,a)*.*

(a) *If*$\frac{2n}{t}$*is even, then*$C_{2n}(a,n)$*is isomorphic to t copies of*$C_{\frac{2n}{t}}(1,\frac{n}{t})$*.*
(b) *If*$\frac{2n}{t}$*is odd, then*$C_{2n}(a,n)$*is isomorphic to*$\frac{t}{2}$*copies of*$C_{\frac{4n}{t}}(2,\frac{2n}{t})$*.*

Remark 1.2 Let $\text{b}=(b_{1},b_{2},\ldots ,b_{2n})$, $b_{i}>0$ for all *i*. If 1≤a<n, and t=gcd⁡(2n,a), then from Theorem 1.1 it follows that

(a) If $\frac{2n}{t}$ is even, then $W(C_{2n}(a,n),\text{b})$ is isomorphic to *t* copies of $W(C_{\frac{2n}{t}}(1,\frac{n}{t}),\text{b})$.
(b) If $\frac{2n}{t}$ is odd, then $W(C_{2n}(a,n),\text{b})$ is isomorphic to $\frac{t}{2}$ copies of $W(C_{\frac{4n}{t}}(2,\frac{2n}{t}),\text{b})$.

It is easy to see that $W(C_{2n}(a,n),\text{b})$ is either a disjoint union of some copies $W(C_{\frac{2n}{t}}(1,\frac{n}{t}),\text{b})$ or $W(C_{\frac{4n}{t}}(2,\frac{2n}{t}),\text{b})$.

**Figure 1:**
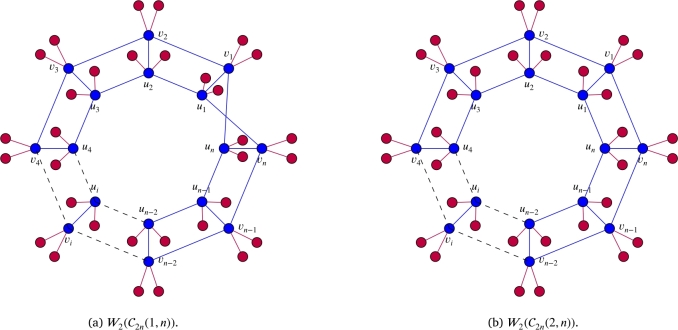
(a) and (b) are the whisker graphs of $C_{2n}(1,n)$ and $C_{2n}(2,n)$, respectively.

It is evident from Remark 1.2 and some known results given in the subsequent section that aforementioned algebraic invariants associated with edge ideals of $W(C_{2n}(a,n),\text{b})$ can be determined by using the algebraic invariants of the edge ideals associated with graphs $W(C_{2n}(1,n),\text{b})$ and $W(C_{2n}(2,n),\text{b})$. See Fig. 1 for examples of these graphs. To find the algebraic invariants of the edge ideals of graphs $W(C_{2n}(1,n),\text{b})$ and $W(C_{2n}(2,n),\text{b})$ we further require the algebraic invariants of some subgraphs that are discussed in section 3. We prove the following theorem for regularity:

Theorem 1.3 *Let*n≥2*,*1≤a<n*,*t=gcd⁡(2n,a)*,*$I=I(W(C_{2n}(a,n),\text{b}))$*and*$Q=F[V(W(C_{2n}(a,n),\text{b}))]$*.*

(a) *If*$\frac{2n}{t}$*is even, then*
  $$\mathrm{reg}(Q/I)=\left\{ \begin{array}{cc} n-t, & \text{if }\frac{n}{t}\text{ is even;}\\ n, & \text{if }\frac{n}{t}\text{ is odd.} \end{array}\right.$$
(b) *If*$\frac{2n}{t}$*is odd, then*
  $$\mathrm{reg}(Q/I)=\frac{2n-t}{2}.$$

We also compute the depth and a lower bound for Stanley depth of the quotient ring of the edge ideal of the graph $W_{q}(C_{2n}(a,n))$:

Theorem 1.4 *Let*n≥2*,*1≤a<n*,*t=gcd⁡(2n,a)*,*$I=I(W_{q}(C_{2n}(a,n)))$*and*$Q=F[V(W_{q}(C_{2n}(a,n)))]$*.*

(a) *If*$\frac{2n}{t}$*is even, then*
  $$\mathrm{sdepth}(Q/I)\geq \mathrm{depth}(Q/I)=\left\{ \begin{array}{cc} n(q+1), & \text{if }\frac{n}{t}\text{ is odd;}\\ n(q+1)+t(q-1), & \text{if }\frac{n}{t}\text{ is even.} \end{array}\right.$$
(b) *If*$\frac{2n}{t}$*is odd, then*
  $$\mathrm{sdepth}(Q/I)\geq \mathrm{depth}(Q/I)=\frac{2n(q+1)+t(q-1)}{2}.$$

We acknowledge the use of CoCoA [8] and Macaulay 2 [14] for our experiments.

## 2 Preliminaries

In this section, we present some definitions and findings that are extensively used in the subsequent sections of this paper. Assume that **N** is a $Z^{m}$-graded *Q*-module which is finitely generated. Let wF[B] represent the F-subspace generated by *wr*, here *w* is a homogeneous element in **N**, $B\subset \{u_{1},u_{2},\ldots ,u_{m}\}$ and *r* is a monomial in F[B]. If wF[B] is a free F[B]-module, then wF[B] is a *Stanley space* of dimension |B|. A decomposition D of *K*-vector space **N** as a finite direct sum of Stanley spaces is called a *Stanley decomposition* of **N**. Let $D:N=\underset{j=1}{\overset{z}{⨁}}w_{j}F[B_{j}]$, the Stanley depth of decomposition D is $\mathrm{sdepth}(D):=\min\{|B_{j}|:j=1,2,\ldots ,z\}$. The *Stanley depth* of **N** is

sdepth(N):=max⁡{sdepth(D):Dis a Stanley decomposition ofN}.

In 1982 Stanley conjectured in [37] that sdepth(N)≥depth(N). In 2016, Duval et al. disproved this conjecture in [12] by giving a counter-example. For more details regarding Stanley depth, we refer the readers to [17], [18], [30], [31]. For many classes of edge ideals of graphs, depth and Stanley depth are discussed; see for instance [5], [21], [22], [43]. Here we recall some known results that are frequently used in this paper.

Lemma 2.1 **[5, Theorem 1.3.3]**

*Let R be a local Noetherian commutative ring and N is a non-zero finitely generated R-module, which has finite projective dimension, then*

depth(R)=pdim(N)+depth(N).

The following lemmas are important to find lower and upper bounds of the depth and Stanley depth of modules.

Lemma 2.2 **[5, Lemma 3.3]**

*Let*I⊂Q*be a squarefree monomial ideal and*$\mathrm{supp}(I)=\{u_{1},u_{2},\ldots ,u_{m}\}$*, suppose*$\omega :=u_{i_{1}}u_{i_{2}}\ldots u_{i_{q}}\in Q/I$*, such that*$u_{l}\omega \in I$*, for all*$u_{l}\in \{u_{1},u_{2},\ldots ,u_{m}\}\setminus \mathrm{supp}(\omega )$*. Then*sdepth(Q/I)≤q*.*

Lemma 2.3 **[5]**

*If*$0\to U^{\prime }\to U\to U^{\prime \prime }\to 0$*is a short exact sequence of modules over a local ring R, or a Noetherian graded ring with*$R_{0}$*local, then*

(a) $\mathrm{depth}(U)\geq \min\{\mathrm{depth}(U^{\prime \prime }),\mathrm{depth}(U^{\prime })\}$*.*
(b) $\mathrm{depth}(U^{\prime })\geq \min\{\mathrm{depth}(U),\mathrm{depth}(U^{\prime \prime })+1\}$*.*
(c) $\mathrm{depth}(U^{\prime \prime })\geq \min\{\mathrm{depth}(U^{\prime })-1,\mathrm{depth}(U)\}$*.*

Lemma 2.4 **[31, Lemma 2.2]**

*Let*$0\to U^{\prime }\to U\to U^{\prime \prime }\to 0$*be a short exact sequence of*$Z^{m}$*-graded Q-module. Then*$\mathrm{sdepth}(U)\geq \min\{\mathrm{sdepth}(U^{\prime }),\mathrm{sdepth}(U^{\prime \prime })\}$*.*

Lemma 2.5 **[40, Proposition 2.2.21]**

*Let*$I_{1}\subset Q^{\prime }=F[u_{1},\ldots ,u_{n}]$*,*$I_{2}\subset Q^{\prime \prime }=F[u_{n+1},\ldots ,u_{m}]$*be monomial ideals, where*1≤q<m*. If*$Q=Q^{\prime }{⊗}_{F}Q^{\prime \prime }$*, then*

$$\mathrm{depth}(Q^{\prime }/I_{1}{⊗}_{F}Q^{\prime \prime }/I_{2})=\mathrm{depth}(Q/(I_{1}Q+I_{2}Q)))={\mathrm{depth}}_{Q^{\prime }}(Q^{\prime }/I_{1})+{\mathrm{depth}}_{Q^{\prime \prime }}(Q^{\prime \prime }/I_{2}).$$

Lemma 2.6 **[11, Lemma 2.13]**

*Let*$I_{1}\subset Q^{\prime }=F[u_{1},\ldots ,u_{n}]$*,*$I_{2}\subset Q^{\prime \prime }=F[u_{n+1},\ldots ,u_{m}]$*be monomial ideal, where*1≤n<m*and*$Q=Q^{\prime }{⊗}_{F}Q^{\prime \prime }$*. Then*

$$\mathrm{sdepth}(Q^{\prime }/I_{1}{⊗}_{F}Q^{\prime \prime }/I_{2})=\mathrm{sdepth}(Q/(I_{1}Q+I_{2}Q))\geq {\mathrm{sdepth}}_{Q^{\prime }}(Q^{\prime }/I_{1})+{\mathrm{sdepth}}_{Q^{\prime \prime }}(Q^{\prime \prime }/I_{2}).$$

Lemma 2.7 **[31, Corollary 1.3] and [7, Proposition 2.7]**

*Let*I⊂Q*be a monomial ideal. Then, for all monomials*u∉I*,*

(a) depth(Q/(I:u))≥depth(Q/I)*.*
(b) sdepth(Q/I)≤sdepth(Q/(I:u))*.*

According to [17, Lemma 3.6], adding a new variable in the polynomial ring leads to an increase in both the depth and Stanley depth, while the regularity remains unaffected [28, Lemma 3.5]. The following lemma presents a concise summary of these results.

Lemma 2.8 *Let*I⊂Q*be a monomial ideal. If*$Q^{\prime }=Q{⊗}_{F}F[u_{m+1}]≅Q[u_{m+1}]$*, then*

(a) $\mathrm{depth}(Q^{\prime }/IQ^{\prime })=\mathrm{depth}(Q/I)+1$*.*
(b) $\mathrm{sdepth}(Q^{\prime }/IQ^{\prime })=\mathrm{sdepth}(Q/I)+1$*.*
(c) $\mathrm{reg}(Q^{\prime }/IQ^{\prime })=\mathrm{reg}(Q/I)$*.*

Let us recall some important results for regularity.

Lemma 2.9 **[6, Theorem 4.7]**

*If*I*be monomial ideal and let u be a variable in Q, then*

(a) reg(Q/I)=1+reg(Q/(I:u))*, if*reg(Q/(I,u))<reg(Q/(I:u))*.*
(b) reg(Q/I)∈{reg(Q/(I,u)),reg(Q/(I,u))+1}*, if*reg(Q/(I:u))=reg(Q/(I,u))*.*
(c) reg(Q/I)=reg(Q/(I,u))*if*reg(Q/(I:u))<reg(Q/(I,u))*.*

Part (a) and part (c) of Lemma 2.9 are proved as Corollary 20.19 and Proposition 20.20, respectively in [13], while part (b) is deduced from Lemma 2.10 of [9]. The following lemma was first proved by Kalai et al. in [23] for squarefree monomial ideals and later on Herzog extended this result to any monomial ideals in [16].

Lemma 2.10 *Let* $I_{1}$ *and* $I_{2}$ *be the monomial ideals of Q, then* $\mathrm{reg}(Q/(I_{1}+I_{2}))\leq \mathrm{reg}(Q/I_{1})+\mathrm{reg}(Q/I_{2})$ *.*

Definition 2.11 Let q≥1. A tree with one internal vertex and *q* leaves attached to it is known as a q−star, denoted as $S_{q}$.

In [36], Shaukat et al. discuss regularity, Stanley depth, and depth of $Q/I(W_{q}(P_{n}))$ and also the regularity of $Q/I(S_{q})$. Alipour et al. in [1], discuss the values of depth and Stanley depth of $Q/I(S_{q})$.

Lemma 2.12 **[36, Lemma 2.2 and Lemma 2.26]**

*Let*q≥2*,*$J=I(S_{q})$*and*$Q=F[V(S_{q})]$*. Then*

depth(Q/J)=reg(Q/J)=sdepth(Q/J)=1.

Lemma 2.13 **[36, Theorem 2.28]**

*Let* $J=I(W_{q}(P_{n}))$ *and* $Q=F[V(W_{q}(P_{n}))]$ *. Then*

(a) $\mathrm{reg}(Q/J)=\lceil \frac{n}{2}\rceil$ *.*
(b) $\mathrm{depth}(Q/J)=\mathrm{sdepth}(Q/J)=\lceil \frac{n}{2}\rceil +\lceil \frac{n-1}{2}\rceil q$ *.*

## 3 Algebraic invariants of cyclic modules associated with some subgraphs of the whisker graphs of $C_{2n}(1,n)$ and $C_{2n}(2,n)$

**Figure 2:**
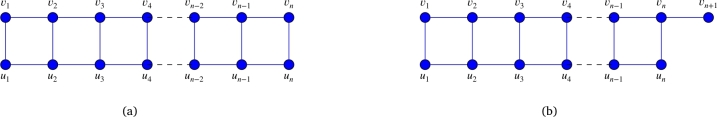
(a) is the graph *E*_*n*_ and (b) is the graph *F*_*n*_.

**Figure 3:**
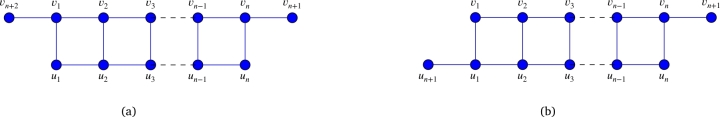
(a) is the graph *G*_*n*_ and (b) is the graph *J*_*n*_.

**Figure 4:**
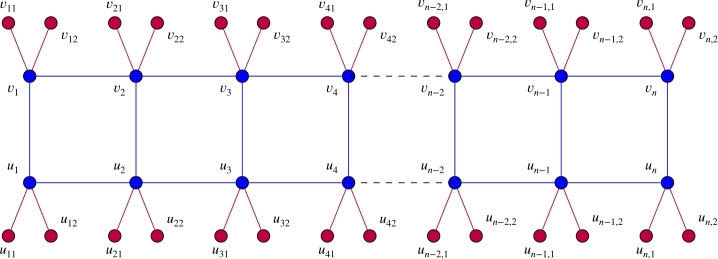
*W*_2_(*E*_*n*_).

For n≥1, let $E_{n}$, $F_{n}$, $G_{n}$, and, $J_{n}$ be graphs as shown in Fig. 2 and Fig. 3. In this section, we give values for the regularity of the residue class rings of the edge ideals $I(W(E_{n},\text{b}))$, $I(W(F_{n},\text{b}))$, $I(W(G_{n},\text{b}))$ and $I(W(J_{n},\text{b}))$. We also compute projective dimension, depth, and Stanley depth of the residue class ring of $I(W_{q}(J_{n}))$. Example of $W_{2}(E_{n})$ is given in Fig. 4. The results of this section are instrumental for the last section of this paper, where we will prove our main results.

A *subgraph F* of a graph *G* is a graph in which the vertex set of *F* is the subset of the vertex set of *G* and the edge set of *F* is the subset of the edge set of *G*. Let D⊆V(G), an *induced subgraph* of *G* is a graph $G^{\prime }:=(D,E(G^{\prime }))$, where $E(G^{\prime })=\{\{u_{i},u_{j}\}\in E(G):\{u_{i},u_{j}\}\subseteq D\}$. In a graph *G*, a *matching*, denoted by *M*, is a subset of E(G) where each pair of edges do not share a common vertex. An *induced matching* in *G* refers to a matching that forms an induced subgraph of *G*. The *induced matching number* of a graph *G* is represented by indmat(G) and is defined as follows:

indmat(G)=max⁡{|M|:M is an induced matching in G}.

A simple graph *G* is called *bipartite graph* if its vertex set can be partitioned into two separate subsets, that is, $V=V_{1}⋃V_{2}$ such that every edge in the graph connects a vertex from $V_{1}$ to a vertex in $V_{2}$. Furthermore, a bipartite graph is called *complete bipartite graph* if every vertex of $V_{1}$ is connected to every vertex of $V_{2}$.

Definition 3.1 Let *G* be a graph and $e_{1}=\{u_{1},v_{1}\}$, $e_{2}=\{u_{2},v_{2}\}$ be two distinct edges in *G*. Then $e_{1}$ and $e_{2}$ are called 3-disjoint if $\{u_{1},v_{1}\}\cap \{u_{2},v_{2}\}=\emptyset$ and $e_{1}\cup e_{2}$ is an induce subgraph on $\left\{u_{1},v_{1},u_{2},v_{2}\right\}$. A subset $\left\{e_{1},e_{2},\ldots ,e_{r}\right\}$ of E(G) is said to be pairwise 3-disjoint subset if $e_{i}$, $e_{j}$ are 3-disjoint, 1≤i, j≤r (i≠j). Moreover, the induced matching number of a graph *G* is the largest size of the pairwise 3-disjoint subset of E(G).

In [24, Lemma 2.2], Katzman showed that reg(Q/I(G)) is bounded below by the induced matching number of *G*, and later Hà et al. showed in [15, Corollary 6.9] that for a chordal graph *G* the indmat(G) is equal to the regularity of Q/I(G). These results are combined in the following lemma.

Lemma 3.2 *If G is a graph, then,*reg(Q/I(G))≥indmat(G)*. Moreover, if G is a chordal graph, then*reg(Q/I(G))=indmat(G)

Definition 3.3 A graph *G* is *weakly chordal graph* if neither *G* nor its complement graph $G^{c}$ contain an induced cycle of length n≥5.

Lemma 3.4 **[42, Theorem 14]**

*For a weakly chordal graph G,* reg(Q/I(G))=indmat(G) *.*

Lemma 3.5 **[19, Lemma 3.2]**

*If* $I_{1}\subset Q^{\prime }=F[u_{1},\ldots ,u_{n}]$ *and* $I_{2}\subset Q^{\prime \prime }=F[u_{n+1},\ldots ,u_{m}]$ *are non-zero homogeneous ideals of* $Q^{\prime }$ *and* $Q^{\prime \prime }$ *and regard* $I_{1}+I_{2}$ *as a homogeneous ideal of* $Q=Q^{\prime }{⊗}_{F}Q^{\prime \prime }$ *, then*

$$\mathrm{reg}(Q/I_{1}+I_{2})=\mathrm{reg}(Q^{\prime }/I_{1})+\mathrm{reg}(Q^{\prime \prime }/I_{2}).$$

Theorem 3.6 *Let G be a graph. Then* W(G,b) *is weakly chordal if and only if G is weakly chordal.*

Proof Suppose *G* is a weakly chordal graph, then both *G* and $G^{c}$ have no induced cycles of length greater than 4. The graph W(G,b) is obtained by adding whiskers to every vertex of *G*. The addition of these whiskers does not introduce any new induced cycle with lengths greater than 4 in W(G,b). Now we show that $W{\left(G,\text{b}\right)}^{c}$ also has no induced cycle of length greater than 4. Since $G^{c}$ is an induced subgraph of $W{\left(G,\text{b}\right)}^{c}$, therefore, every cycle of length greater than 4 involving the vertices of $G^{c}$ in $W{\left(G,\text{b}\right)}^{c}$ has a chord. Now if $v\in V(W{\left(G,\text{b}\right)}^{c})$ such that *v* is not a vertex of the induced subgraph $G^{c}$ of $\left(W{\left(G,\text{b}\right)}^{c}\right)$, then *v* is adjacent to all other vertices of $W{\left(G,\text{b}\right)}^{c}$. This proves that $W{\left(G,\text{b}\right)}^{c}$ has no induced cycle of length greater than 4. Conversely; assume that W(G,b) is a weakly chordal graph, thus both W(G,b) and $W{\left(G,\text{b}\right)}^{c}$ do not contain cycles of length greater than 4. Since *G* and $G^{c}$ are induced subgraphs of W(G,b) and $W{\left(G,\text{b}\right)}^{c}$, respectively, therefore both *G* and $G^{c}$ have no induced cycles of length greater than 4. □

Let *G* be a graph and S⊂V(G), *S* is called an *independent set* if no two vertices in *S* are adjacent in *G*. A *maximum independent set* is an independent set of the largest possible size. The cardinality of the maximum independent set is called *independence number* of *G* and is denoted by α(G).

Theorem 3.7 *If G is a graph, then* indmat(W(G,b))=α(G) *.*

Proof Let $\left\{u_{1},u_{2},\ldots ,u_{\alpha (G)}\right\}$ be a maximum independent set in *G*. Let $b_{i}$ be a whisker to $u_{i}$ in W(G,b) then clearly the set $\left\{\{u_{1},b_{1}\},\{u_{2},b_{2}\},\ldots ,\{u_{\alpha (G)},b_{\alpha (G)}\}\right\}$ is an induced matching of W(G,b). This shows that α(G)≤indmat(W(G,b)). Now we prove the other inequality. Let indmat(W(G,b))=n and $B=\{e_{1},e_{2},\ldots ,e_{n}\}$ be the corresponding induce matching of W(G,b). One can assume that one vertex in each $e_{i}\in B$ is a whisker to a vertex of the graph *G*. Indeed, if there exist an edge $e_{i}=\{u_{i},v_{i}\}\in B$ such that $u_{i},v_{i}\in V(G)$, then one can replace the edge $\left\{u_{i},v_{i}\right\}$ in *B* with $\left\{u_{i},b_{i}\right\}$ (where $b_{i}$ is a whisker to $u_{i}$) and *B* is still an induced matching. Thus we have $B=\{\{u_{1},b_{1}\},\{u_{2},b_{2}\},\ldots ,\{u_{n},b_{n}\}\}$ (where $u_{i}\in V(G)$), it follows that $\left\{u_{1},u_{2},\ldots ,u_{n}\right\}$ is an independent set of *G*. Thus indmat(W(G,b))≤α(G). By combining these two inequalities we get indmat(W(G,b))=α(G). □

Lemma 3.8 *Let* n≥2 *and* $Q=F[V(W(P_{n},\text{b}))]$ *. Then* $\mathrm{reg}(Q/I(W(P_{n},\text{b})))=\lceil \frac{n}{2}\rceil$ *.*

Proof Let $P_{n}$ be the path graph of the vertex set $\left\{u_{1},u_{2},\ldots ,u_{n}\right\}$. The maximum independent set of $P_{n}$ contains the alternative vertices of $P_{n}$. That means the size of the maximum independent set is $\left\lceil \frac{n}{2}\right\rceil$. Since the path graph is a weakly chordal graph, then by Theorem 3.7, $W(P_{n},\text{b})$ is also a weakly chordal graph, thus by Lemma 3.4, $\mathrm{reg}(W(P_{n},\text{b}))=\lceil \frac{n}{2}\rceil$. □

Let $A_{i}:=\{u_{i1},u_{i2},\ldots ,u_{im_{i}}\}$ be the set of pendant vertices attached to $u_{i}$ and $A_{i}^{\prime }:=\{v_{i1},v_{i2},\ldots ,v_{in_{i}}\}$ be the set of pendant vertices attached to $v_{i}$.

Lemma 3.9 *Let* n≥1 *,* $Q=F[V(W(E_{n},\text{b}))]$ *and* $I=I(W(E_{n},\text{b}))$ *. Then* reg(Q/I)=n *.*

Proof Consider a subset $L=\{u_{1},v_{2},u_{3},v_{4},\ldots ,u_{n-1},v_{n}\}$ of $V(E_{n})$ when *n* is even. And for case when *n* is odd, $L=\{u_{1},v_{2},u_{3},v_{4},\ldots ,v_{n-1}u_{n}\}$. One can easily see that *L* is a maximum independent set of $E_{n}$ and |L|=n. Since $E_{n}$ is a weakly chordal graph then by Theorem 3.6, $W(E_{n},\text{b})$ is a weakly chordal graph. Thus, by applying Theorem 3.7, reg(Q/I)=n. □

Lemma 3.10 *Let* n≥1 *,* $Q=F[V(W(F_{n},\text{b}))]$ *and* $I=I(W(F_{n},\text{b}))$ *. Then* reg(Q/I)=n+1 *.*

Proof The proof of this Lemma is similar to the proof of Lemma 3.9. But the maximum independent set of $F_{n}$ is $L=\{u_{1},v_{2},u_{3},v_{4},\ldots ,u_{n},v_{n+1}\}$ when *n* is odd. For the case when *n* is even, $L=\{v_{1},u_{2},v_{3},u_{4},\ldots ,u_{n}v_{n+1}\}$. □

Lemma 3.11 *Let* n≥1 *,* $Q=F[V(W(G_{n},\text{b}))]$ *and* $I=I(W(G_{n},\text{b}))$ *. Then*

$$\mathrm{reg}(Q/I)=\left\{ \begin{array}{cc} n+1, & \text{if}\;\text{n}\;\text{is even;}\\ n+2, & \text{if}\;\text{n}\;\text{is odd.} \end{array}\right.$$

Proof Again, the proof of this Lemma is similar to the proof of Lemma 3.9. If *n* is odd, we take $L=\{u_{1},v_{2},u_{3},v_{4},\ldots ,v_{n-1},u_{n},v_{n+1},v_{n+2}\}$. For the case when *n* is even we take $L=\{u_{1},v_{2},u_{3},v_{4},\ldots ,u_{n-1},v_{n},v_{n+2}\}$. □

Lemma 3.12 *Let* n≥1 *,* $Q=F[V(W(J_{n},\text{b}))]$ *and* $I=I(W(J_{n},\text{b}))$ *. Then*

$$\mathrm{reg}(Q/I)=\left\{ \begin{array}{cc} n+2, & \text{if}\;\text{n}\;\text{is even;}\\ n+1, & \text{if}\;\text{n}\;\text{is odd.} \end{array}\right.$$

Proof Similarly we follow the proof of Lemma 3.9. If *n* is odd we consider

$$L=\{u_{1},v_{2},u_{3},v_{4},\ldots ,v_{n-1},u_{n},v_{n+1}\},$$

and if *n* is even we consider $L=\{v_{1},u_{2},v_{3},u_{4},\ldots ,v_{n-1},u_{n},v_{n+1},u_{n+1}\}$. □

Definition 3.13 **[25]**

A family $B=\{B_{1},B_{2},\ldots ,B_{\gamma }\}$ of complete bipartite subgraphs of *G* is defined to be strongly disjoint if it satisfies the following conditions:

(a) $V(B_{\alpha })⋂V(B_{\beta })=\emptyset$, for 1≤α, β≤γ(α≠β).
(b) Additionally, there is an induced matching $\left\{e_{1},e_{2},\ldots ,e_{\gamma }\right\}$ of *G*, such that $e_{\alpha }\in E(B_{\alpha })$, for 1≤α≤γ.

Lemma 3.14 **[29, Theorem 7.7]**

*Let G be a weakly chordal graph (G has no isolated vertices) then* pdim(F[V(G)]/I(G))=d(G) *, where* $d(G)=\max\{\sum_{\alpha =1}^{\gamma }|V(B_{\alpha })|-\gamma \}$ *, with the maximum taken over all strongly disjoint collections* $\left\{B_{1},B_{2},\ldots ,B_{\gamma }\right\}$ *of complete bipartite subgraphs of G.*

Lemma 3.15 *Let* n,q≥1 *. Then*

$$d(W_{q}(J_{n}))=\left\{ \begin{array}{cc} q(n+2)+n, & \text{if}\;\text{n}\;\text{is even;}\\ (n+1)(q+1), & \text{if}\;\text{n}\;\text{is odd.} \end{array}\right.$$

Proof We consider the following cases:

**Case 1**: Let *n* be odd. If $B_{1}=\{u_{1}u_{n+1},u_{1}u_{11},\ldots ,u_{1}u_{1q}\}$, $B_{2}=\{v_{2}v_{1},v_{2}v_{21},\ldots ,v_{2}v_{2q}\}$, …, $B_{n}=\{u_{n}u_{n-1},u_{n}u_{n1},\ldots ,u_{n}u_{nq}\}$, and $B_{n+1}=\{v_{n+1}v_{n-1},v_{n+1}v_{(n+1)1},\ldots ,v_{n+1}v_{(n+1)q}\}$, then one can easily see that $B=\{B_{1},B_{2},\ldots ,B_{n+1}\}$ is the family of strongly disjoint complete bipartite subgraphs of $J_{n,q}$. Moreover, if $C=\{C_{1},C_{2},\ldots ,C_{r}\}$ is any strongly disjoint family of complete bipartite subgraph of $W_{q}(J_{n})$ it is easy to see that $\sum_{\alpha =1}^{n+1}|V(B_{\alpha })|-(n+1)\geq \sum_{\beta =1}^{r}|V(C_{\beta })|-r$. Thus $d(W_{q}(J_{n}))=\sum_{\alpha =1}^{n+1}|V(B_{\alpha })|-(n+1)=(q+2)(n+1)-(n+1)=(n+1)(q+1)$.
**Case 2**: Let *n* be even. If $B_{1}=\{u_{n+1}u_{1},u_{n+1}u_{(n+1)1},\ldots ,u_{n+1}u_{(n+1)q}\}$, $B_{2}=\{v_{1}v_{2},v_{1}v_{11},\ldots ,v_{1}v_{1q}\}$, …, $B_{n}=\{v_{n-1}v_{n},v_{n-1}v_{(n-1)1},\ldots ,v_{n-1}v_{(n-1)q}\}$, $B_{n+1}=\{u_{n}u_{n1},\ldots ,u_{n}u_{nq}\}$, and $B_{n+2}=\{v_{n+1}v_{(n+1)1},\ldots ,v_{n+1}v_{(n+1)q}\}$, then clearly, $B=\{B_{1},B_{2},\ldots ,B_{n+2}\}$ is a family of strongly disjoint complete bipartite subgraphs of $J_{n,q}$. Moreover, if $C=\{C_{1},C_{2},\ldots ,C_{r}\}$ is any strongly disjoint family of complete bipartite subgraph of $W_{q}(J_{n})$, then
  $$\sum_{\alpha =1}^{n+2}|V(B_{\alpha })|-(n+2)\geq \sum_{\beta =1}^{r}|V(C_{\beta })|-r.$$
  In this case, one can see that the $|V(B_{1})|=|V(B_{2})|=\ldots =|V(B_{n})|=q+2$ and $|B_{n+1}|=|B_{n+2}|=q+1$. Thus $d(W_{q}(J_{n}))=\{\sum_{\alpha =1}^{n+2}|V(B_{\alpha })|-(n+2)\}=(q+2)(n)+2(q+1)-(n+2)=q(n+2)+n$. □

Proposition 3.16 *Let* q,n≥1 *,* $Q=F[V(W_{q}(J_{n}))]$ *and* $I=I(W_{q}(J_{n}))$ *. Then*

$$\mathrm{pdim}(Q/I)=\left\{ \begin{array}{cc} q(n+2)+n, & \text{if}\;\text{n}\;\text{is even;}\\ (n+1)(q+1), & \text{if}\;\text{n}\;\text{is odd.} \end{array}\right.$$

Proof Since $W_{q}(J_{n})$ is a weakly chordal graph, so by Lemma 3.14 and Lemma 3.15,

$$\mathrm{pdim}(Q/I)=\left\{ \begin{array}{cc} q(n+2)+n, & \text{if}\;\text{n}\;\text{is even;}\\ (n+1)(q+1), & \text{if}\;\text{n}\;\text{is odd.} \end{array}\right.$$

 □

Theorem 3.17 *Let* q,n≥1 *,* $Q=F[V(W_{q}(J_{n}))]$ *and* $I=I(W_{q}(J_{n}))$ *. Then*

$$\mathrm{depth}(Q/I)=\left\{ \begin{array}{cc} n(q+1)+2, & \text{if}\;\text{n}\;\text{is even;}\\ (n+1)(q+1), & \text{if}\;\text{n}\;\text{is odd.} \end{array}\right.$$

Proof The desired outcome can be obtained by applying Lemma 2.1 and Lemma 3.16. □

Wang et al. proved the below results for depth and Stanley depth in [41].

Lemma 3.18 **[41, Theorem 3]**

*If*n,q≥1*and*$Q=F[V(W_{q}(E_{n}))]$*, then*

$$\mathrm{depth}(Q/I(W_{q}(E_{n})))=\mathrm{sdepth}(Q/I(W_{q}(E_{n})))=(q+1)n.$$

Lemma 3.19 **[41, Lemma 8]**

*If* n,q≥1 *and* $Q=F[V(W_{q}(F_{n}))]$ *, then*

$$\mathrm{depth}(Q/I(W_{q}(F_{n})))=\mathrm{sdepth}(Q/I(W_{q}(F_{n})))=n(q+1)+1.$$

Remark 3.20 For $W_{q}(J_{n})$, we discuss some special cases. If n=0, then

$$F[V(W_{q}(J_{0}))]/I(W_{q}(J_{0}))≅F[V(S_{q})]/I(S_{q}){⊗}_{F}F[V(S_{q})]/I(S_{q}).$$

Thus by Lemma 2.6,

$$\mathrm{sdepth}(F[V(W_{q}(J_{0}))]/I(W_{q}(J_{0})))\geq \mathrm{sdepth}(F[V(S_{q})]/I(S_{q}))+\mathrm{sdepth}(F[V(S_{q})]/I(S_{q}))$$

and by applying Lemma 2.12, $\mathrm{sdepth}(F[V(W_{q}(J_{0}))]/I(W_{q}(J_{0})))\geq 2$. If n=1, then

$$F[V(W_{q}(J_{1}))]/I(W_{q}(J_{1}))≅F[V(W_{q}(P_{4}))]/I(W_{q}(P_{4}))$$

so by Lemma 2.13

$$\mathrm{sdepth}(F[V(W_{q}(J_{1}))]/I(W_{q}(J_{1})))=\mathrm{sdepth}(F[V(W_{q}(P_{4}))]/I(W_{q}(P_{4})))=2(1+q).$$

Lemma 3.21 *Let* q,n≥1 *,* $Q=F[V(W_{q}(J_{n}))]$ *and* $I=I(W_{q}(J_{n}))$ *. Then*

$$\mathrm{sdepth}(Q/I)=\left\{ \begin{array}{cc} n(q+1)+2, & \text{if}\;\text{n}\;\text{is even;}\\ (n+1)(q+1), & \text{if}\;\text{n}\;\text{is odd.} \end{array}\right.$$

Proof Consider the following short exact sequence

(1) $$0⟶Q/(I:u_{n})\overset{\cdot u_{n}}{\to }Q/I⟶Q/(I,u_{n})⟶0,$$

we have

(2) $$Q/(I:u_{n})≅F[V(W_{q}(J_{n-2}))]/I(W_{q}(J_{n-2})){⊗}_{F}F[V(S_{q})]/I(S_{q}){⊗}_{F}F[\{u_{n}\}\cup A_{n-1}\cup A_{n}^{\prime }].$$

Let $L:=(I,u_{n})=(I(W_{q}(J_{n-1})),u_{n},v_{n}v_{n+1},v_{n+1}v_{(n+1)1},v_{n+1}v_{(n+1)2},\ldots ,v_{n+1}v_{(n+1)q})$, and consider the short exact sequence

(3) $$0⟶Q/I(L:v_{n+1})\overset{\cdot v_{n+1}}{\to }S/I(L)⟶Q/I(L,v_{n+1})⟶0.$$

We have the following isomorphisms:

(4) $$Q/I(L:v_{n+1})≅F[V(W_{q}(F_{n-1}))]/I(W_{q}(F_{n-1})){⊗}_{F}F[\{v_{n+1}\}\cup A_{n}^{\prime }\cup A_{n}],$$

(5) $$Q/I(L,v_{n+1})≅F[V(W_{q}(J_{n-1}))]/I(W_{q}(J_{n-1})){⊗}_{F}F[A_{n+1}^{\prime }\cup A_{n}].$$

Let n=1. Then by Remark 3.20, $\mathrm{sdepth}(F[V(W_{q}(J_{1}))]/(I(W_{q}(J_{1}))))\geq 2q+2$. For the second inequality, suppose $\omega =u_{11}\ldots u_{1q}v_{21}\ldots v_{2q}v_{1}u_{2}\in F[V(W_{q}(J_{1}))]/I(W_{q}(J_{1}))$. For all $u\in \mathrm{supp}(I(W_{q}(J_{1})))\setminus \mathrm{supp}(\omega )$, clearly uω∈I thus by applying Lemma 2.2, $\mathrm{sdepth}(F[V(W_{q}(J_{1}))]/I(W_{q}(J_{1}))\leq 2q+2$. Thus, $\mathrm{sdepth}(F[V(W_{q}(J_{1}))]/I(W_{q}(J_{1}))=2q+2$.

Let n≥2, then we have the following scenarios:

**Case 1**: If *n* is odd, then by using Lemma 2.6 and Lemma 2.8 on Eqs. (2), (4) and (5), respectively,
  $$\mathrm{sdepth}(Q/(I:u_{n}))\geq \mathrm{sdepth}(F[V(W_{q}(J_{n-2}))]/I(W_{q}(J_{n-2})))+\mathrm{sdepth}[(F(V(S_{q}))]/I(S_{q}))+1+q+q,$$
  $$\mathrm{sdepth}(Q/(L:v_{n+1}))=\mathrm{sdepth}(F[V(W_{q}(F_{n-1}))]/I(W_{q}(F_{n-1})))+1+q+q,$$
  $$\mathrm{sdepth}(Q/(L,v_{n+1}))=\mathrm{sdepth}(F[V(W_{q}(J_{n-1}))]/I(W_{q}(J_{n-1})))+q+q.$$
  Applying the principle of mathematical induction on *n*, along with the use of Lemma 2.12 and Lemma 3.19,
  (6) $$\mathrm{sdepth}(Q/(I:u_{n}))\geq n(q+1)-q-1+1+1+2q\geq (n+1)(q+1),$$
  (7) $$\mathrm{sdepth}(Q/(L:v_{n+1}))=(n+1)(q+1),$$
  (8) $$\mathrm{sdepth}(Q/(L,v_{n+1}))=(n+1)(q+1).$$
  By applying Lemma 2.4 and Lemma 2.7 on Eqs. (7) and (8), respectively, along with the use of short exact sequence (3)
  (9) $$\mathrm{sdepth}(Q/(I,u_{n}))=(n+1)(q+1).$$
  Using Lemma 2.4 on Eqs. (6) and (9), respectively along with the use of short exact sequence (1), sdepth(Q/I)≥(n+1)(q+1). For the second inequality, let
  $$\omega =u_{11}\ldots u_{1q}v_{21}\ldots v_{2q}\ldots v_{(n-1)1}\ldots v_{(n-1)q}u_{n1}\ldots u_{nq}v_{(n+1)1}\ldots v_{(n+1)q}v_{1}u_{2}\ldots u_{n-1}v_{n}u_{n+1}\in Q/I.$$
  Clearly uω∈I, thus, by applying Lemma 2.2, sdepth(Q/I)≤(q+1)(n+1). Thus, sdepth(Q/I)=(q+1)(n+1).
**Case 2**: If *n* is even, then for
  $$\omega =u_{11}\ldots u_{1q}v_{21}\ldots v_{2q}\ldots u_{(n-1)1}\ldots u_{(n-1)q}v_{n1}\ldots v_{nq}v_{(n+1)1}\ldots v_{(n+1)q}v_{1}u_{2}\ldots v_{n-1}u_{n}u_{n+1}\in Q/I,$$
  and proceeding on the same lines as Case 1, we have sdepth(Q/I)=n(q+1)+2. □

## 4 Algebraic invariants of cyclic modules associated with whisker cubic circulant graphs

**Figure 5:**
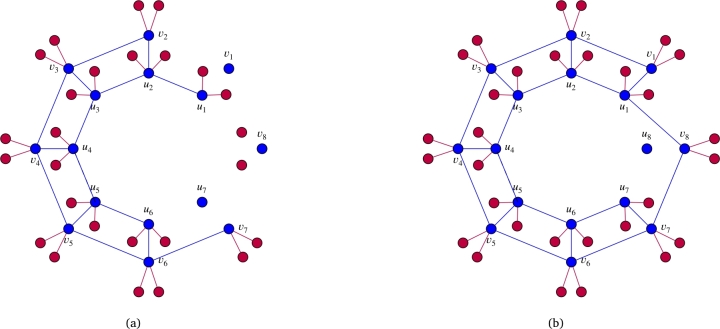
(a) represents graph $G_{\left(I(W_{2}(C_{16}(1,8))):u_{8}\right)}$ and (b) represents $G_{\left(I(W_{2}(C_{16}(1,8))),u_{8}\right)}$.

**Figure 6:**
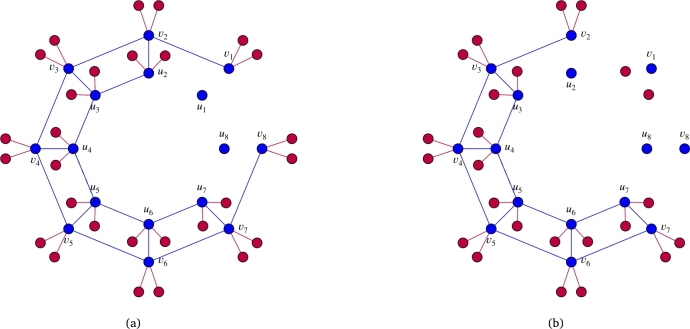
(a) represents graph $G_{\left((I(W_{2}(C_{16}(1,8))),u_{8}),u_{1}\right)}$ and (b) represents graph $G_{\left((I(W_{2}(C_{16}(1,8))),u_{8}):u_{1}\right)}$.

In this section, we compute Stanley depth, depth, and projective dimension of the quotient rings of the edge ideals of $W_{q}(C_{2n}(a,n))$ along with the regularity of $W(C_{2n}(a,n),\text{b})$. Furthermore, we also calculate depth, Stanley depth, and projective dimension of the quotient rings of the edge ideals of $W_{q}(C_{2n}(1,n))$, and determine the regularity of the quotient rings of the edge ideals of $W(C_{2n}(2,n),\text{b})$ and $W(C_{2n}(1,n),\text{b})$. Let I be a square free monomial ideal generated by monomials of degree at most 2. Let $G_{I}$ be the graph associated to I with $V(G_{I})=\mathrm{supp}(I)$ and $E(G_{I})=\{\{u_{i},u_{j}\}:u_{i}u_{j}\in G(I)\}$. To provide better insight into the proof strategy, we present the following example before proving the main results. See for instance; Fig. 5 and Fig. 6 as examples of $G_{\left(I(W_{2}(C_{16}(1,8))):u_{8}\right)}$, $G_{\left(I(W_{2}(C_{16}(1,8))),u_{8}\right)}$, $G_{\left((I(W_{2}(C_{16}(1,8))),u_{8}),u_{1}\right)}$ and $G_{\left((I(W_{2}(C_{16}(1,8))),u_{8}):u_{1}\right)}$. Using Fig. 5 and Fig. 6, it is easy to understand the following isomorphisms:

$$F[V(W_{2}(C_{16}(1,8)))]/(I(W_{2}(C_{16}(1,8)):u_{8}))≅F[V(W_{2}(J_{5}))]/I(W_{2}(J_{5})){⊗}_{F}F[\{u_{8}\}\cup A_{8}^{\prime }\cup A_{7}\cup A_{1}^{\prime }],$$

$$F[V(W_{2}(C_{16}(1,8)))]/(I(W_{2}(C_{16}(1,8))),u_{8})≅F[V(W_{2}(E_{7})),v_{8}]/(I(W_{2}(E_{7})),u_{1}v_{8},v_{7}v_{8},v_{8}v_{81},v_{8}v_{82}){⊗}_{F}F[A_{8}],$$

$$F[V(W_{2}(C_{16}(1,8)))]/((I(W_{2}(C_{16}(1,8))),u_{8}),u_{1})≅F[V(W_{2}(G_{6}))]/I(W_{2}(G_{6})){⊗}_{F}F[A_{8}\cup A_{1}],$$

$$F[V(W_{2}(C_{16}(1,8)))]/((I(W_{2}(C_{16}(1,8))),u_{8}):u_{1})≅F[V(W_{2}(F_{5}))]/I(W_{2}(F_{5})){⊗}_{F}F[\{u_{1}\}\cup A_{8}^{\prime }\cup A_{1}^{\prime }\cup A_{2}\cup A_{8}].$$

Theorem 4.1 *Let* n≥3 *,* $Q=F[V(W(C_{2n}(2,n),\text{b}))]$ *, and* $I=I(W(C_{2n}(2,n),\text{b})))$ *. Then*

$$\mathrm{reg}(Q/I)=\left\{ \begin{array}{cc} n, & \text{if}\;\text{n}\;\text{is even;}\\ n-1, & \text{if}\;\text{n}\;\text{is odd.} \end{array}\right.$$

Proof If n=3, then

$$Q/(I:u_{3})≅F[V(W(P_{2},\text{b}))]/I(W(P_{2},\text{b})){⊗}_{F}F[\{u_{3}\}\cup A_{3}^{\prime }\cup A_{2}\cup A_{1}],$$

$$Q/((I,u_{3}):v_{2})≅F[V(S_{q})]/I(S_{q}){⊗}_{F}F[\{v_{2}\}\cup A_{3}\cup A_{1}^{\prime }\cup A_{3}^{\prime }\cup A_{2}],$$

$$Q/((I,u_{3}),v_{2})≅F[V(W(J_{1},\text{b}))]/I(W(J_{1},\text{b})){⊗}_{F}F[A_{2}^{\prime }\cup A_{3}^{\prime }].$$

Applying Lemma 2.8, $\mathrm{reg}(Q/(I:u_{3}))=\mathrm{reg}(F[V(W(P_{2},\text{b}))]/I(W(P_{2},\text{b})))$,

$$\mathrm{reg}(Q/((I,u_{3}):v_{2}))=\mathrm{reg}(F[V(S_{q})]/I(S_{q})),$$

$$\mathrm{reg}(Q/((I,u_{3}),v_{2}))=\mathrm{reg}(F[V(W(J_{1},\text{b}))]/I(W(J_{1},\text{b}))).$$

Using Lemma 2.12 and Lemma 3.8, $\mathrm{reg}(Q/(I:u_{3}))=1$, $\mathrm{reg}(Q/((I,u_{3}):v_{2}))=1$. By applying Lemma 3.12,

$$\mathrm{reg}(Q/((I,u_{3}),v_{2}))=2.$$

Since

$$\mathrm{reg}(Q/((I,u_{3}):v_{2}))<\mathrm{reg}(Q/((I,u_{3}),v_{2})),$$

so by Lemma 2.9(c), $\mathrm{reg}(Q/(I,u_{3}))=2>\mathrm{reg}(Q/(I:u_{3}))$. Again by applying Lemma 2.9(c), reg(Q/I)=2. Let n≥4. We will now examine the two scenarios:

**Case 1**: Let *n* be an odd. Then applying Lemma 2.9(c), $\mathrm{reg}(Q/I)=\mathrm{reg}(Q/(I,u_{n}))$, if
  $$\mathrm{reg}(Q/(I:u_{n}))<\mathrm{reg}(Q/(I,u_{n})).$$
  Since
  $$Q/(I:u_{n})≅F[V(W(G_{n-3},\text{b}))]/I(W(G_{n-3},\text{b})){⊗}_{F}F[\{u_{n}\}\cup A_{n}^{\prime }\cup A_{1}\cup A_{n-1}],$$
  $$Q/(I,u_{n}):v_{n-1})≅F[V(W(F_{n-3},\text{b}))]/I(W(F_{n-3},\text{b})){⊗}_{F}F[\{v_{n-1}\}\cup A_{n}\cup A_{n-2}^{\prime }\cup A_{n}^{\prime }\cup A_{n-1}],$$
  $$Q/((I,u_{n}),v_{n-1})≅F[V(W(J_{n-2},\text{b}))]/I(W(J_{n-2},\text{b})){⊗}_{F}F[A_{n}\cup A_{n-1}^{\prime }].$$
  Applying Lemma 2.8, $\mathrm{reg}(Q/((I:u_{n}))=\mathrm{reg}(F[V(W(G_{n-3},\text{b}))]/I(W(G_{n-3},\text{b})))$,
  $$\mathrm{reg}(Q/((I,u_{n}):v_{n-1}))=\mathrm{reg}(F[V(W(F_{n-3},\text{b}))]/I(W(F_{n-3},\text{b}))),$$
  $$\mathrm{reg}(Q/((I,u_{n}),v_{n-1}))=\mathrm{reg}(F[V(W(J_{n-2},\text{b}))]/I(W(J_{n-2},\text{b}))).$$
  Using Lemma 3.11 and Lemma 3.10,
  $$\mathrm{reg}(Q/(I:u_{n}))=n-2,\;\text{and}\;\mathrm{reg}(Q/((I,u_{n}):v_{n-1}))=n-2.$$
  By Lemma 3.12, $\mathrm{reg}(Q/((I,u_{n}),v_{n-1}))=n-1$.
  As, $\mathrm{reg}(Q/((I,u_{n}):v_{n-1}))<\mathrm{reg}(Q/((I,u_{n}),v_{n-1}))$, by using Lemma 2.9(c),
  $$\mathrm{reg}(Q/(I,u_{n}))=n-1.$$
  Also $\mathrm{reg}(Q/(I:u_{n}))<\mathrm{reg}(Q/(I,u_{n}))$, again by applying Lemma 2.9(c),
  reg(Q/I)=n−1.
**Case 2**: Let *n* be an even. Then $W(C_{2n}(2,n),\text{b}))≅W(E_{n-1},\text{b})⋃W(P_{2},\text{b})$, where $W(E_{n-1},\text{b})⋂W(P_{2},\text{b})\neq \emptyset$. In $W(P_{2},\text{b})$ at least two pendants attached with the vertices of $E_{1}$. By applying Lemma 2.10 and Lemma 3.9,
  $$\mathrm{reg}(Q/I)\leq \mathrm{reg}(F[V(W(E_{n-1},\text{b}))]/I(W(E_{n-1},\text{b})))+\mathrm{reg}(F[V(W(P_{2},\text{b}))]/I(W(P_{2},\text{b})))=n.$$
  Regarding the second inequality, the maximum independent set of $C_{2n}(2,n)$ is $\left\{u_{1},v_{2},\ldots ,u_{n-1},v_{n}\right\}$, then by Theorem 3.7, induce matching number of $W(C_{2n}(2,n),\text{b})$ is *n*. By applying Lemma 3.2, reg(Q/I)≥n. Therefore, reg(Q/I)=n. □

Theorem 4.2 *If* n≥3 *,* $Q=F[V(W(C_{2n}(1,n),\text{b}))]$ *, and* $I=I(W(C_{2n}(1,n),\text{b}))$ *then*

$$\mathrm{reg}(Q/I)=\left\{ \begin{array}{cc} n-1, & \text{if}\;\text{n}\;\text{is even;}\\ n, & \text{if}\;\text{n}\;\text{is odd.} \end{array}\right.$$

Proof If n=3, then

$$Q/(I:u_{3})≅F[V(S_{q})]/I(S_{q}){⊗}_{F}F[\{u_{3}\}\cup A_{3}^{\prime }\cup A_{2}\cup A_{1}^{\prime }],$$

$$Q/((I,u_{3}),u_{1})≅F[V(W(G_{1},\text{b}))]/I(W(G_{1},\text{b})){⊗}_{F}F[A_{3}\cup A_{1}],$$

$$Q/((I,u_{3}):u_{1})≅F[V(S_{q})]/I(S_{q}){⊗}_{F}F[\{u_{1}\}\cup A_{3}\cup A_{1}^{\prime }\cup A_{2}\cup A_{3}^{\prime }].$$

By applying Lemma 2.12 and Lemma 2.8,

$$\mathrm{reg}(Q/(I:u_{3}))=\mathrm{reg}(F[V(S_{q})]/I(S_{q}))=1,$$

$$\mathrm{reg}(Q/((I,u_{3}):u_{1}))=\mathrm{reg}(F[V(S_{q})]/I(S_{q}))=1.$$

Using Lemma 3.11 and Lemma 2.8,

$$\mathrm{reg}(Q/((I,u_{3}),u_{1}))=\mathrm{reg}(F[V(W(G_{1},\text{b}))]/I(W(G_{1},\text{b})))=3.$$

Since, $\mathrm{reg}(Q/((I,u_{3}):u_{1}))<\mathrm{reg}(Q/((I,u_{3}),u_{1}))$. By using Lemma 2.9(c),

$$Q/(I,u_{3})=3>Q/(I:u_{3}).$$

Again by Lemma 2.9(c),

reg(Q/I)=3.

Let n≥4. We will now examine the two scenarios:

**Case 1**: Let *n* be an odd. Then by applying Lemma 2.9(c), $\mathrm{reg}(Q/I)=\mathrm{reg}(Q/(I,u_{n}))$, if
  $$\mathrm{reg}(Q/(I:u_{n}))<\mathrm{reg}(Q/(I,u_{n})).$$
  We have:
  $$Q/(I:u_{n})≅F[V(W(J_{n-3},\text{b}))]/I(W(J_{n-3},\text{b})){⊗}_{F}F[\{u_{n}\}\cup A_{n}^{\prime }\cup A_{n-1}\cup A_{1}^{\prime }],$$
  $$Q/((I,u_{n}):u_{1})≅F[V(W(F_{n-3},\text{b}))]/I(W(F_{n-3},\text{b})){⊗}_{F}F[\{u_{1}\}\cup A_{n}^{\prime }\cup A_{n-1}\cup A_{1}^{\prime }],$$
  $$Q/((I,u_{n}),u_{1})≅F[V(W(G_{n-2},\text{b}))]/I(W(G_{n-2},\text{b})){⊗}_{F}F[A_{n}\cup A_{1}].$$
  Now using Lemma 2.8, $\mathrm{reg}(Q/(I:u_{n}))=\mathrm{reg}(F[V(W(J_{n-3},\text{b}))]/I(W(J_{n-3},\text{b})))$,
  $$\mathrm{reg}(Q/((I,u_{n}):u_{1}))=\mathrm{reg}(F[V(W(F_{n-3},\text{b}))]/I(W(F_{n-3},\text{b}))),$$
  $$\mathrm{reg}(Q/((I,u_{n}),u_{1}))=\mathrm{reg}(F[V(W(G_{n-2},\text{b}))]/I(W(G_{n-2},\text{b}))).$$
  By applying Lemma 3.10 and Lemma 3.12, $\mathrm{reg}(Q/(I:u_{n}))=n-1$, $\mathrm{reg}(Q/((I,u_{n}):u_{1}))=n-2$. By Lemma 3.11,
  $$\mathrm{reg}(Q/((I,u_{n}),u_{1}))=n.$$
  As, $\mathrm{reg}(Q/((I,u_{n}):u_{1}))<\mathrm{reg}(Q/((I,u_{n}),u_{1}))$, by applying Lemma 2.9(c),
  $$\mathrm{reg}(Q/(I,u_{n}))=n.$$
  Also, $\mathrm{reg}(Q/(I:u_{n}))<\mathrm{reg}(Q/(I,u_{n}))$, by applying Lemma 2.9(c), reg(Q/I)=n.
**Case 2**: Let *n* be an even. Then by the same strategy as case 1, one can easily see that reg(Q/I)=n−1. □

Lemma 4.3 **[41, Theorem 6]**

*Let* n≥3 *,* q≥1 *,* $Q=F[V(W_{q}(C_{2n}(2,n)))]$ *, and* $I=I(W_{q}(C_{2n}(2,n)))$ *. Then*

$$\mathrm{sdepth}(Q/I)=\mathrm{depth}(Q/I)=\left\{ \begin{array}{cc} n(q+1), & \text{if}\;\text{n}\;\text{is even;}\\ n(q+1)+q-1, & \text{if}\;\text{n}\;\text{is odd.} \end{array}\right.$$

Remark 4.4 For $W_{q}(F_{n})$, we have a special case for n=0. Let n=0 we have the following isomorphism $F[V(W_{q}(F_{0}))]/I(W_{q}(F_{0}))≅F[V(W_{q}(P_{2}))]/I(W_{q}(P_{2}))$. By Lemma 2.13,

$$\mathrm{depth}(F[V(W_{q}(F_{0}))]/I(W_{q}(F_{0})))=\mathrm{depth}(F[V(W_{q}(P_{2}))]/I(W_{q}(P_{2})))=1.$$

Theorem 4.5 *Let* n≥3 *,* q≥1 *,* $Q=F[V(W_{q}(C_{2n}(1,n)))]$ *, and* $I=I(W_{q}(C_{2n}(1,n)))$ *. Then*

$$\mathrm{sdepth}(Q/I)=\mathrm{depth}(Q/I)=\left\{ \begin{array}{cc} n(q+1)+q-1, & \text{if}\;\text{n}\;\text{is even;}\\ n(q+1), & \text{if}\;\text{n}\;\text{is odd.} \end{array}\right.$$

Proof Firstly, we will apply induction on *n* to compute depth. We have

(10) $$0⟶Q/(I:u_{n})\overset{\cdot u_{n}}{\to }Q/I⟶Q/(I,u_{n})⟶0.$$

Then,

(11) $$Q/(I:u_{n})≅F[V(W_{q}(J_{n-3}))]/I(W_{q}(J_{n-3})){⊗}_{F}F[\{u_{n}\}\cup A_{n-1}\cup A_{n}^{\prime }\cup A_{1}^{\prime }].$$

$$L:=(I,u_{n})=(I(W_{q}(F_{n-1})),u_{n},u_{1}v_{n},A_{n}).$$

Again

(12) $$0⟶Q/I(L:v_{n})\overset{\cdot v_{n}}{\to }Q/I(L)⟶Q/I(L,v_{n})⟶0.$$

(13) $$Q/(L:v_{n})≅F[V(W_{q}(J_{n-3}))]/I(W_{q}(J_{n-3})){⊗}_{F}F[\{v_{n}\}\cup A_{n-1}^{\prime }\cup A_{1}\cup A_{n}],$$

(14) $$Q/(L,v_{n})≅F[V(W_{q}(E_{n-1}))]/I(W_{q}(E_{n-1})){⊗}_{F}F[A_{n}\cup A_{n}^{\prime }].$$

If n=3, then it is an easy exercise to prove that $\mathrm{depth}(F[V(W_{q}(C_{2n}(1,3)))]/I(W_{q}(C_{2n}(1,3))))=3(q+1)$. Suppose n≥4, now we have two scenarios:

**Case 1**: If *n* is odd, then by applying Lemma 2.5 and Lemma 2.8 on Eq. (11),
  (15) $$\mathrm{depth}(Q/(I:u_{n}))=\mathrm{depth}(F[V(W_{q}(J_{n-3}))]/I(W_{q}(J_{n-3})))+1+3q.$$
  Now by Lemma 3.17 on Eq. (15),
  (16) $$\mathrm{depth}(Q/(I:u_{n}))=n(q+1).$$
  Applying Lemma 2.5 and Lemma 2.8 on Eqs. (13) and (14), respectively
  $$\mathrm{depth}(Q/(L:v_{n}))=\mathrm{depth}(F[V(W_{q}(J_{n-3}))]/I(W_{q}(J_{n-3})))+1+3q,$$
  $$\mathrm{depth}(Q/(L,v_{n}))=\mathrm{depth}(F[V(W_{q}(E_{n-1}))]/I(W_{q}(E_{n-1})))+q+q.$$
  By using Lemma 3.18 and Lemma 3.17, $\mathrm{depth}(Q/(L:v_{n}))=n(q+1)$, $\mathrm{depth}(Q/(L,v_{n}))=n(q+1)+q-1$.
  Using Lemma 2.3 and Lemma 2.7 along with the use of short exact sequence (12),
  (17) depth(Q/L)=n(q+1).
  Applying Lemma 2.3 and Lemma 2.7 on Eqs. (16) and (17), respectively along with the use of short exact sequence (10), depth(Q/I)=n(q+1).
**Case 2**: Let *n* is even. As case 1, it follows that depth(Q/I)=n(q+1)+q−1.
  Now we will find Stanley's depth. Let n=3. Then, by a similar method as depth, just use Lemma 2.4 in place of Lemma 2.3 and also use 3.21 instead of 3.17. We obtain, $\mathrm{sdepth}(Q/(I(W_{q}(C_{2n}(1,3)))))\geq 3(q+1)$. For the second inequality, let
  $$\omega =v_{11}\ldots v_{1q}u_{21}\ldots u_{2q}v_{31}\ldots v_{3q}u_{1}v_{2}v_{2}u_{3}u_{1}u_{3}\in Q/I(W_{q}(C_{2n}(1,3))).$$
  For all $u\in \mathrm{supp}(I(W_{q}(C_{2n}(1,3))))\setminus \mathrm{supp}(\omega )$, clearly $u\omega \in I(W_{q}(C_{2n}(1,3)))$. Thus by applying Lemma 2.2,
  $$\mathrm{sdepth}(Q/I(W_{q}(C_{2n}(1,3))))\leq 3(q+1).$$
  Thus,
  $$\mathrm{sdepth}(Q/I(W_{q}(C_{2n}(1,3))))=3(q+1).$$
  The proof for the Stanley depth for n≥4 is the same as the proof for depth; just use Lemma 2.4 instead of Lemma 2.3. □

Lemma 4.6 *Let* n≥3 *,* q≥1 *,* $Q=F[V(W_{q}(C_{2n}(1,n)))]$ *, and* $I=I(W_{q}(C_{2n}(1,n)))$ *. Then*

$$\mathrm{pdim}(Q/I)=\left\{ \begin{array}{cc} n(q+1)-q+1, & \text{if}\;\text{n}\;\text{is even;}\\ n(q+1), & \text{if}\;\text{n}\;\text{is odd.} \end{array}\right.$$

Proof The desired outcome can be obtained by applying Lemma 2.1 and Lemma 4.5. □

Remark 4.7 Let n≥2, q≥1, t=gcd⁡(2n,a) and 1≤a<n. Note that Remark 1.2 (b) implies that $W(C_{2n}(a,n),\text{b})$ is isomorphic to $\frac{t}{2}$ copies of $W(C_{\frac{4n}{t}}(2,\frac{2n}{t}),\text{b})$. For $W(C_{\frac{4n}{t}}(2,\frac{2n}{t}),\text{b})$, we only need to consider the case when $\frac{2n}{t}$ is odd. If $\frac{2n}{t}$ is even, then by Remark 1.2 (a), we have *t* distinct copies of $W(C_{\frac{2n}{t}}(1,\frac{n}{t}),\text{b})$.

Theorem 4.8 *Let* n≥2 *,* 1≤a<n *,* t=gcd⁡(2n,a) *,* $I=I(W(C_{2n}(a,n),\text{b}))$ *and* $Q=F[V(W(C_{2n}(a,n),\text{b}))]$ *.*

(a) *If* $\frac{2n}{t}$ *is even, then*
  $$\mathrm{reg}(Q/I)=\left\{ \begin{array}{cc} n-t, & \text{if }\frac{n}{t}\text{ is even;}\\ n, & \text{if }\frac{n}{t}\text{ is odd.} \end{array}\right.$$
(b) *If* $\frac{2n}{t}$ *is odd, then*
  $$\mathrm{reg}(Q/I)=\frac{2n-t}{2}.$$

Proof Let $\frac{2n}{t}$ be even. Since t=gcd⁡(2n,a), therefore $\frac{n}{t}\geq 2$ and a positive integer. Now by using Theorem 4.2,

$$\mathrm{reg}(F[V(W(C_{\frac{2n}{t}}(1,\frac{n}{t}),\text{b}))]/I(W(C_{\frac{2n}{t}}(1,\frac{n}{t}),\text{b})))=\left\{ \begin{array}{cc} \frac{n}{t}-1, & \text{if }\frac{n}{t}\text{ is even;}\\ \frac{n}{t}, & \text{if }\frac{n}{t}\text{ is odd.} \end{array}\right.$$

By Remark 1.2, $W(C_{2n}(a,n),\text{b})$ is isomorphic to *t* copies of $W(C_{\frac{2n}{t}}(1,\frac{n}{t}),\text{b})$. By Lemma 3.5,

$$\mathrm{reg}(Q/I)=\left\{ \begin{array}{cc} n-t, & \text{if }\frac{n}{t}\text{ is even;}\\ n, & \text{if }\frac{n}{t}\text{ is odd.} \end{array}\right.$$

Now, if $\frac{2n}{t}$ is odd, then $\frac{2n}{t}>2$ and is a positive integer. Now, by using Theorem 4.1,

$$\mathrm{reg}(F[V(W(C_{\frac{4n}{t}}(2,\frac{2n}{t}),\text{b})]/I(W(C_{\frac{4n}{t}}(2,\frac{2n}{t}),\text{b}))=\frac{2n}{t}-1.$$

By Remark 1.2, $W(C_{2n}(a,n),\text{b})$ is isomorphic to $\frac{t}{2}$ copies of $W(C_{\frac{4n}{t}}(2,\frac{2n}{t}),\text{b})$. By Remark 4.7, it is enough to consider the cases when $\frac{2n}{t}$ is odd; so by Lemma 3.5, the desired result follows

$$\mathrm{reg}(Q/I)=\frac{2n-t}{2}.$$

 □

Theorem 4.9 *Let* n≥2 *,* 1≤a<n *,* t=gcd⁡(2n,a) *,* $I=I(W_{q}(C_{2n}(a,n)))$ *and* $Q=F[V(W_{q}(C_{2n}(a,n)))]$ *.*

(a) *If* $\frac{2n}{t}$ *is even, then*
  $$\mathrm{sdepth}(Q/I)\geq \mathrm{depth}(Q/I)=\left\{ \begin{array}{cc} n(q+1), & \text{if }\frac{n}{t}\text{ is odd;}\\ n(q+1)+t(q-1), & \text{if }\frac{n}{t}\text{ is even.} \end{array}\right.$$
(b) *If* $\frac{2n}{t}$ *is odd, then*
  $$\mathrm{sdepth}(Q/I)\geq \mathrm{depth}(Q/I)=\frac{2n(q+1)+t(q-1)}{2}.$$

Proof Let $\frac{2n}{t}$ be even. Since t=gcd⁡(2n,a), therefore $\frac{n}{t}\geq 2$ and a positive integer. Now by using Theorem 4.5,

$$\mathrm{depth}(F[V(W_{q}(C_{\frac{2n}{t}}(1,\frac{n}{t})))]/I(W_{q}(C_{\frac{2n}{t}}(1,\frac{n}{t}))))=\left\{ \begin{array}{cc} \frac{n(q+1)}{t}, & \text{if }\frac{n}{t}\text{ is odd;}\\ \frac{n(q+1)}{t}+q-1, & \text{if }\frac{n}{t}\text{ is even.} \end{array}\right.$$

$$\mathrm{sdepth}(F[V(W_{q}(C_{\frac{2n}{t}}(1,\frac{n}{t})))]/I(W_{q}(C_{\frac{2n}{t}}(1,\frac{n}{t}))))=\left\{ \begin{array}{cc} \frac{n(q+1)}{t}, & \text{if }\frac{n}{t}\text{ is odd;}\\ \frac{n(q+1)}{t}+q-1, & \text{if }\frac{n}{t}\text{ is even.} \end{array}\right.$$

By Remark 1.2, $W_{q}(C_{2n}(a,n))$ is isomorphic to *t* copies of $W_{q}(C_{\frac{2n}{t}}(1,\frac{n}{t}))$. By Lemma 2.5 (for depth) and Lemma 2.6 (for Stanley depth),

$$\mathrm{sdepth}(Q/I)\geq \mathrm{depth}(Q/I)=\left\{ \begin{array}{cc} n(q+1), & \text{if }\frac{n}{t}\text{ is odd;}\\ n(q+1)+t(q-1), & \text{if }\frac{n}{t}\text{ is even.} \end{array}\right.$$

Now, if $\frac{2n}{t}$ is odd, then $\frac{2n}{t}>2$ and is a positive integer. By using Theorem 4.3, we have

$$\mathrm{depth}(F[V(W_{q}(C_{\frac{4n}{t}}(2,\frac{2n}{t})))]/I(W_{q}(C_{\frac{4n}{t}}(2,\frac{2n}{t}))))=\frac{2n}{t}(q+1)+q-1.$$

$$\mathrm{sdepth}(F[V(W_{q}(C_{\frac{4n}{t}}(2,\frac{2n}{t})))]/I(W_{q}(C_{\frac{4n}{t}}(2,\frac{2n}{t}))))\geq \frac{2n}{t}(q+1)+q-1.$$

By Remark 1.2, $W_{q}(C_{2n}(a,n))$ is isomorphic to $\frac{t}{2}$ copies of $W_{q}(C_{\frac{4n}{t}}(2,\frac{2n}{t}))$. By Remark 4.7, it is enough to consider the cases when $\frac{2n}{t}$ is odd; so by Lemma 2.5 (for depth) and Lemma 2.6 (for Stanley depth), the desired result follows

$$\mathrm{sdepth}(Q/I)\geq \mathrm{depth}(Q/I)=\frac{2n(q+1)+t(q-1)}{2}.$$

 □

Corollary 4.10 *Let* n≥2 *,* 1≤a<n *,* t=gcd⁡(2n,a) *,* $I=I(W_{q}(C_{2n}(a,n)))$ *and* $Q=F[V(W_{q}(C_{2n}(a,n)))]$ *.*

(a) *If* $\frac{2n}{t}$ *is even,*
  $$\mathrm{pdim}(Q/I)=\left\{ \begin{array}{cc} n(1+q), & \text{if }\frac{n}{t}\text{ is odd;}\\ n(1+q)-t(q-1), & \text{if }\frac{n}{t}\text{ is even.} \end{array}\right.$$
(b) *If* $\frac{2n}{t}$ *is odd,*
  $$\mathrm{pdim}(Q/I)=\frac{2n(1+q)-t(q-1)}{2}.$$

Proof The desired result can be obtained by applying Lemma 2.1 and Lemma 4.9. □

## CRediT authorship contribution statement

**Mujahid Ullah Khan Afridi:** Writing – original draft, Validation, Methodology, Investigation. **Ibad Ur Rehman:** Writing – original draft, Validation, Methodology, Investigation. **Muhammad Ishaq:** Writing – review & editing, Supervision, Conceptualization.

## Declaration of Competing Interest

The authors declare that they have no known competing financial interests or personal relationships that could have appeared to influence the work reported in this paper.

## Data Availability

Has data associated with your study been deposited into a publicly available repository? No. Has data associated with your study been deposited into a publicly available repository? No data was used for the research described in the article.
